# Excess weight and its association with cardiometabolic risk in adolescents: comparative analysis of national and international classifications

**DOI:** 10.1590/1984-0462/2026/44/2025342

**Published:** 2026-10-19

**Authors:** Maria Dinara de Araújo Nogueira, Luis Felipe Nunes de Oliveira, Géssica de Souza Martins, Ribanna Aparecida Marques Braga, Eric Wenda Ribeiro Lourenço, Ilana Nogueira Bezerra, Carla Soraya Costa Maia

**Affiliations:** a Universidade Estadual do Ceará, Centro de Ciências da Saúde, Programa de Pós-Graduação em Saúde Coletiva, Fortaleza, CE, Brazil.; b Universidade Estadual do Ceará, Health Sciences Center, Centro de Ciências da Saúde, Programa de Pós-Graduação em Nutrição e Saúde, Fortaleza, CE, Brazil.; c Universidade de São Paulo, Faculdade de Medicine, Instituto do Coração, Programa de Pós-Graduação em Cardiologia, São Paulo, SP, Brazil.; d Universidade Estadual do Ceará, Centro de Ciências da Saúde, Fortaleza, CE, Brazil.

**Keywords:** Adolescent health, Nutritional status, Anthropometry, Cardiometabolic risk factors, Saúde do adolescente, Estado nutricional, Antropometria, Fatores de risco cardiometabólico

## Abstract

**Objective:**

To evaluate the agreement between the overweight and obesity classifications of the World Health Organization (WHO), the International Obesity Task Force (IOTF), and the Brazilian Reference (BR) proposed by Conde and Monteiro in adolescents, and to verify the association of these classifications with cardiometabolic risk (CMR).

**Methods:**

A cross-sectional study was conducted with 823 adolescents (aged 10-19 years) from municipal schools in Fortaleza, Ceará. Nutritional status was classified according to three body mass index references. CMR was defined by the simultaneous presence of insulin resistance, identified by the waist-to-height ratio, and dyslipidemia, assessed by biochemical tests (total cholesterol, HDL-c, LDL-c, and triglycerides). Agreement between classifications was assessed using the Kappa (κ) statistic, and the association with CMR was analyzed using Poisson regression.

**Results:**

All classifications showed excellent agreement, with weighted κ values ranging from 0.85 to 0.95. The highest agreement was between WHO and IOTF references (κ=0.95; 95%CI 0.93-0.97), while the lowest was between WHO and BR (κ=0.85; 95%CI 0.82-0.88). Despite the lower agreement, the diagnosis of obesity according to the BR reference demonstrated strong clinical relevance, being associated with an 8.51-fold higher prevalence of CMR in adolescents (95%CI 6.38-11.35).

**Conclusions:**

BR showed a stronger association with CMR. The inclusion of this classification may optimize the early detection of CMR in Brazilian health services, helping to prevent cardiometabolic disorders in this population.

## RESUMO

**Objetivo:**

Avaliar a concordância entre as classificações de sobrepeso e obesidade da Organização Mundial da Saúde (OMS), da International Obesity Task Force (IOTF) e da Referência Brasileira (RB) de Conde e Monteiro em adolescentes e verificar a associação dessas classificações com o risco cardiometabólico (RCM).

**Métodos:**

Trata-se de um estudo transversal realizado com 823 adolescentes (10-19 anos) de escolas municipais de Fortaleza, Ceará. O estado nutricional foi classificado segundo as três referências do índice de massa corporal. O RCM foi definido pela presença simultânea de resistência à insulina, identificada pela relação cintura-estatura, e de dislipidemia, avaliada por exames bioquímicos (colesterol total, HDL-c, LDL-c e triglicerídeos). A concordância entre as classificações foi avaliada pelo teste Kappa (κ), e a associação com o RCM foi analisada por meio de regressão de Poisson.

**Resultados:**

Todas as classificações apresentaram excelente concordância, com valores de κ ponderado entre 0,85 e 0,95. A maior concordância foi entre as referências da OMS e da IOTF (κ=0,95; IC95% 0,93-0,97), enquanto a menor foi entre a OMS e a RB (κ=0,85; IC95% 0,82-0,88). Apesar da menor concordância, o diagnóstico de obesidade pela RB demonstrou forte relevância clínica, estando associado a uma prevalência de RCM 8,51 vezes maior em adolescentes (IC95% 6,38-11,35).

**Conclusões:**

A RB apresentou maior associação com o RCM. A inclusão dessa classificação pode otimizar a detecção precoce do RCM nos serviços de saúde brasileiros, auxiliando na prevenção de agravos cardiometabólicos nessa população.

## INTRODUCTION

Excess weight, which includes being overweight and obesity, is one of the major global public health concerns.^1^ In Brazil, the prevalence of overweight among adolescents is high, affecting 16.4% of females and 15.3% of males, while obesity affects 6.2 and 6.7%, respectively.^2^ This condition is associated with risk factors for chronic diseases from adolescence through adulthood, highlighting the need for early prevention of comorbidities such as cardiovascular diseases.^3^

Cardiovascular diseases are the leading cause of global mortality, accounting for 44% of deaths from chronic noncommunicable diseases.^4^ In this context, having excess weight during adolescence is a key risk factor associated with increased cardiometabolic morbidity and premature death. In addition to anthropometric changes, it promotes systemic dysregulation and a chronic low-grade inflammatory state, contributing to increased cardiovascular risk, as evidenced by elevated cardiometabolic risk (CMR) markers, such as dyslipidemia (DLP), hypertension, insulin resistance (IR), and abdominal obesity.^5^ ^,^ ^6^

Given this scenario, early identification of excess weight and CMR in adolescence is essential for targeting effective therapeutic interventions aimed at reducing body weight and restoring metabolic balance, thereby mitigating the risk of future adverse events. To this end, the use of standardized and accurate references for the diagnosis of overweight and obesity is essential for the adequate screening of adolescent populations vulnerable to developing cardiometabolic diseases.^7^

Body mass index-for-age (BMI-for-age) is a practical tool for adolescent nutritional screening due to its low cost and correlation with body fat.^8^ Systems such as the World Health Organization (WHO),^9^ International Obesity Task Force (IOTF),^10^ and the Brazilian Reference (BR)^11^ are commonly used, though they differ in rationale and cut-offs. While the WHO^9^ uses standard deviations, the IOTF^10^ and BR^11^ apply adult thresholds (, the IO^2^ and y adult ^2^) to age-specific curves, the former using multi-country data^10^ and the latter based on the Brazilian population.^11^ Such variability underscores the need for comparative analyses to establish diagnostic standards.^7^ It is hypothesized that the BR classification shows stronger associations with CMR. Thus, this study aimed to evaluate the agreement between WHO, IOTF, and BR classifications and their associations with CMR in adolescents.

## METHOD

This was a cross-sectional study conducted using data from the Dyslipidemia and Dietary Practices in Adolescents in Fortaleza project, collected between April and December 2015, in public schools in Fortaleza, Ceará. It was approved by the Ethics Committee of Hospital Infantil Albert Sabin (approval number 327.850; CAAE: 00907312.5.0000.5042) and followed Resolution 466/12 and the Strengthening the Reporting of Observational Studies in Epidemiology (STROBE) statement.

The study population comprised adolescents enrolled in public schools in Fortaleza in 2015. Adolescents aged 10-19 years, of both sexes, who signed or had their legal guardian sign the informed consent form were included. Adolescents who were not fasting for 12 hours at the time of blood collection and pregnant adolescents were not considered. Subsequently, participants presenting incomplete or inconsistent anthropometric and/or biochemical data in the database were excluded, as well as adolescents with hereditary dyslipidemia.

The target population consisted of 65,415 adolescents. For sample size calculation, a prevalence of 15% for DLP^12^ was adopted among students, derived from the primary outcome of the original study, a sampling error of 3%, and a 5% significance level. Thus, the minimum required sample was calculated to be 775 adolescents. To mitigate the impact of potential dropouts, a 20% margin was added, bringing the final selected sample to 930 participants, who were stratified by regional executive secretariat. The detailed sampling process and full methodology are described in previous publications.^5^ ^,^ ^13^

The variables of interest for this study were categorized to ensure epidemiological rigor. The primary outcome for the present analysis was the presence of CMR, defined by the simultaneous occurrence of IR and DLP. The main exposure was nutritional status, classified using three different systems. Anthropometric measurements [height, weight, and waist circumference (WC)] and biochemical parameters [total cholesterol (TC), high-density lipoprotein cholesterol (HDL-c), triglycerides (TG), and low-density lipoprotein cholesterol (LDL-c)] served as the primary predictors and components of the diagnostic criteria.

Anthropometric measurements were conducted by trained researchers using calibrated equipment, according to the standardized procedures recommended by Lohman et al..^14^ Height was measured using a portable stadiometer (Sanny®) with a range of 2.0 m and a precision of 0.1 cm, and body weight was measured using a portable electronic scale (Seca®) with a capacity of 150 kg and an accuracy of 0.1 kg.

For the assessment of nutritional status, BMI was calculated, and information on sex and age was obtained, as required for the classification of overweight and obesity established by WHO,^9^ IOTF,^10^ and the BR proposed by Conde and Monteiro.^11^ According to WHO, overweight was defined as BMI-for-age >P85 and gP97, and obesity as BMI-for-age >P97.^9^ For the IOTF^10^ and the BR, overweight and obesity were defined using age and sex-specific BMI cutoff points equivalent to BMI values of 25 kg/m^2^ and 30 kg/m^2^, respectively, at 18 years of age.

WC was measured using an inelastic measuring tape positioned at the midpoint between the last rib and the iliac crest. Based on WC, the waist-to-height ratio (WHtR) was calculated as WHtR=WC (cm)/height (cm).

Blood samples for biochemical analyses were collected after a 12-hour fast, in accordance with guidelines provided before the collection day. Analyses of TC, HDL-c, and TG were conducted using standard methods with commercial kits (Roche Diagnosis®) in an automated spectrophotometer (Roche Diagnostics®) at the LEG Clinical Analysis Laboratory (Santa Casa de Misericórdia de Fortaleza, Ceará). LDL-c concentration was calculated using the equation proposed by Friedewald, Levy, and Fredrickson.^15^

The WHtR was used to identify IR associated with abdominal obesity in adolescents, according to the cut-off points established in the Brazilian Diabetes Society Guidelines.^16^ The cut-off points indicated as anthropometric markers of IR are ≥0.475 for girls and ≥0.489 for boys, originally proposed for adolescents aged 10-15 years. As these guidelines do not provide specific cut-off points for individuals aged 16-19 years, the same classification was applied to ensure methodological standardization.

The presence of DLP was defined when at least one of the following alterations was observed: elevated TC (ally pg/dL), elevated LDL-c ( (as defdL), elevated TG ( (( (asdL), or low HDL-c ( ( (as dL), as proposed by the Practical Update Guide on Dyslipidemia of the Brazilian Society of Pediatrics.^17^ CMR was considered present when IR and DLP occurred simultaneously.

Normality and homogeneity of numerical variables were assessed using the Kolmogorov-Smirnov and Levene’s tests, respectively. Categorical variables were described as absolute and relative frequencies, and numerical variables as means and standard deviations (SD).

The relationship between anthropometric variables and cardiometabolic profile, stratified by sex, was analyzed using Student’s *t*-test for independent samples or the Mann-Whitney test, depending on the data distribution. For categorical variables, the association with sex was analyzed using Pearson’s ꭓ^2^ test.

Weighted Kappa (κ) was used to assess the agreement among the definitions of nutritional status. The comparison groups were without excess weight *vs*. overweight *vs*. obesity. The agreement was classified according to the method proposed by Svanholm et al.^18^ as poor (≤0.20), fair (0.20-0.40), moderate (0.41-0.60), good (0.61-0.80), and excellent (>0.80).

Poisson regression was used to evaluate the association between the independent variable (nutritional status in three categories: without excess weight, overweight, and obesity) and the dependent variable (presence of CMR) for each classification. Both unadjusted and model-adjusted for sex and age (potential confounders) were analyzed, and their prevalence ratios (PR) and 95% confidence intervals (95%CI) were reported.

All analyses were performed using Stata software, version 17.0, considering statistical significance at p<0.05.

## RESULTS

The participant selection process for the present study is detailed in Figure 1. After the application of rigorous screening and exclusion criteria, some participants were removed from the sample due to inconsistent or incomplete anthropometric and/or biochemical data. This step led to the formation of the final analytical sample. It is important to note that no analyses were conducted for participants with missing data, thereby ensuring the integrity of the results obtained.

**Figura 1.**
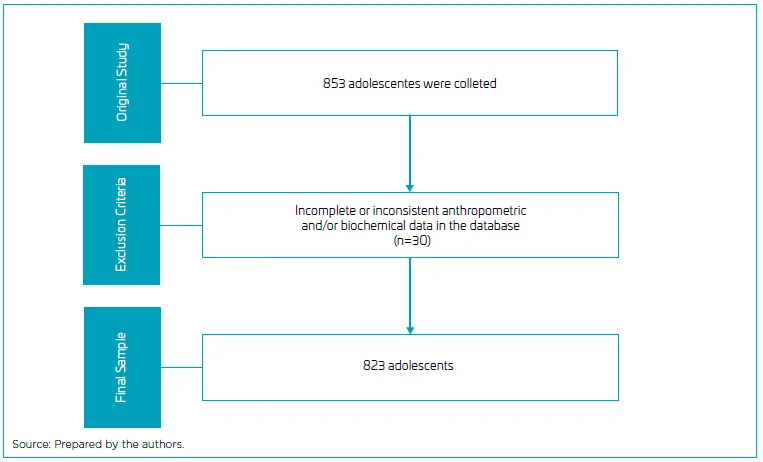
Flowchart of the sample selection process

The mean age of the adolescents evaluated was 13.08 (SD 1.35) years, and most were female (56.62%). A significant difference between sexes was identified only for height, IR, TC, and CMR. All anthropometric and cardiometabolic profile variables are presented in Table 1.

**Table 1.** Anthropometric characteristics and cardiometabolic profile of adolescents according to sex (n=823).

Variables	Total	Female	Male	p-value
	n=823	n=466	n=357	
Age, years, mean (SD)	13.08 (1.35)	13.12 (1.38)	13.02 (1.32)	0.291*
Weight, kg, mean (SD)	50.33 (12.65)	49.88 (11.80)	50.93 (13.66)	0.603^†^
Height, m, mean (SD)	1.55 (0.09)	1.54 (0.07)	1.57 (0.11)	**<0.001^†^**
BMI, kg/m², mean (SD)	20.64 (3.98)	20.80 (3.93)	20.44 (4.04)	0.208*
WC, cm, mean (SD)	73.22 (10.07)	73.04 (9.65)	73.45 (10.61)	0.568*
WtHR, mean (SD)	0.47 (0.06)	0.47 (0.06)	0.46 (0.06)	0.236*
IR by WtHR, n (%)	302 (36.70)	202 (43.30)	100 (28.00)	**<0.001^‡^**
TC, mg/dL, mean (SD)	147.38 (28.27)	149.95 (29.79)	144.03 (25.81)	0.003*
LDL-c, mg/dL, mean (SD)	84.97 (22.17)	86.07 (21.91)	83.55 (22.46)	0.107*
HDL-c, mg/dL, mean (SD)	46.17 (18.71)	47.23 (22.88)	44.78 (10.98)	0.063*
TG, mg/dL, mean (SD)	84.06 (44.38)	86.37 (44.97)	81.04 (43.48)	0.088*
DLP, n (%)	545 (66.20)	311 (66.70)	234 (65.50)	0.720^‡^
CMR, n (%)	225 (27.30)	145 (31.10)	80 (22.40)	**0.005^‡^**

SD: standard deviation; n: frequency; %: percentage; BMI: body mass index; WHtR: waist-to-height ratio; WC: waist circumference; IR: insulin resistance; TC: total cholesterol; LDL-c: low-density lipoprotein; HDL-c: high-density lipoprotein; TG: triglycerides; DLP: dyslipidemia; CMR: cardiometabolic risk (established by the presence of combined DLP and IR).

*Student’s *t*-test for independent samples; ^†^Mann-Whitney test (numerical variables); ^‡^Pearson’s ꭓ^2^ test (categorical variables);

Statistical significance (p<0.05) is indicated in bold.

Source: Prepared by the authors.

The prevalence of excess weight was higher when adolescents were classified according to the BR (33.5%), whereas the WHO and IOTF classifications showed similar prevalences (25.5 and 26.9%, respectively). Regarding obesity, the BR and IOTF classifications presented higher prevalences (9.5 and 8.4%, respectively), while the lowest prevalence was observed with the WHO classification (6.3%). When considering CMR, a larger number of cases occurred among overweight adolescents across all classifications, with the highest number identified for BR (50.7%). The highest proportion of CMR among adolescents without excess weight was observed according to the WHO (36.0%) and IOTF (34.2%) classifications, whereas the BR showed the highest proportion of CMR in the obesity category (27.1%) (Figure 2).

**Figura 2.**
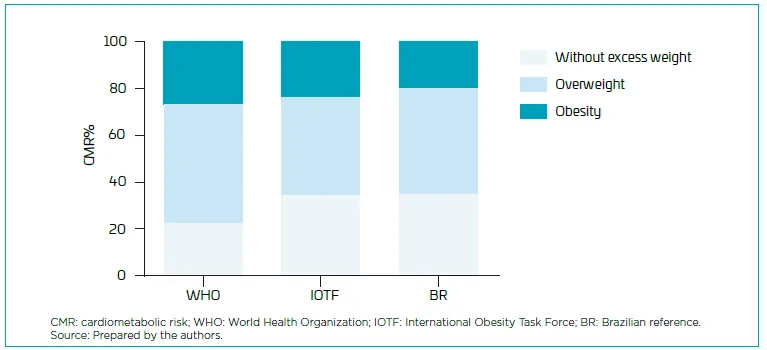
Relative proportion of adolescents with cardiometabolic risk across nutritional classification categories of body-mass index for age/gender, according to the classification systems.

The agreement in the classification of overweight and obesity was excellent across all references evaluated. The highest level of agreement was found between the WHO and IOTF, which presented a weighted κ of 0.95 and a raw agreement of 99% across nutritional status categories. Although showing slightly lower weighted Kappa values, the BR also demonstrated excellent agreement with both the IOTF and WHO (0.89 and 0.85, respectively) (Table 2).

**Table 2.** Agreement between World Health Organization, International Obesity Task Force, and Brazilian reference references for overweight and obesity classification in adolescents, according to the weighted kappa coefficient.

Comparison of references	**Without excess weight *vs*. Overweight *vs*. Obesity**	
	Weighted kappa (95%CI)	Agreement %
WHO x IOTF	0.95 (0.93-0.97)	99
WHO x BR	0.85 (0.82-0.88)	97
BR x IOTF	0.89 (0.86-0.92)	98

WHO: World Health Organization; IOTF: International Obesity Task Force; BR: Brazilian reference.

Source: Prepared by the authors.

All classifications of overweight and obesity were associated with CMR, regardless of sex or age. Adolescents diagnosed as overweight or obese had higher prevalences of CMR compared to those classified as having without excess weight across all classification systems. Among the three systems, the BR classification stood out. Adolescents diagnosed as overweight or obese according to the BR reference had a CMR PR of 7.07 (95%CI 5.37-9.29) and 8.51-fold (95%CI 6.38-11.35) higher, respectively (Table 3).

**Table 3.** Prevalence ratio and confidence interval of the presence of cardiometabolic risk according to classification of without excess weight, overweight, and obesity for adolescents (n=823).

Variables	WHO	IOTF	BR
	PR (95%CI)	PR (95%CI)	PR (95%CI)
No adjustment			
Without excess weight	1	1	1
Overweight	5.19 (4.15-6.48)	5.36 (4.27-6.74)	6.94 (5.25-9.17)
Obesity	6.55 (5.15-8.33)	6.15 (4.83-7.85)	8.55 (6.41-11.42)
Adjusted*			
Without excess weight	1	1	1
Overweight	5.22 (4.18-6.51)	5.37 (4.28-6.74)	7.07 (5.37-9.29)
Obesity	6.93 (5.41-8.87)	6.25 (4.89-8.00)	8.51 (6.38-11.35)

WHO: World Health Organization; IOTF: International Obesity Task Force; BR: Brazilian reference; PR: prevalence ratio; 95%CI: 95% confidence interval.

*Model adjusted for sex and age.

Source: Prepared by the authors.

## DISCUSSION

The results demonstrated excellent agreement among all classifications evaluated, with particular emphasis on the near-perfect agreement between the WHO and IOTF. All classifications were significantly associated with CMR among the adolescents assessed; however, the BR classification showed the highest prevalence ratios for both overweight and obesity, when considered separately.

The excellent agreement among all classification systems used to diagnose the nutritional status of adolescents can be explained by the methodology employed in the development of these tools. The similarity between the IOTF and BR derives from the classification system adopted by both, as they are based on an epidemiological model that uses adult nutritional status as the reference. Furthermore, the IOTF classification incorporates data from the Brazilian population along with information from five other countries, which may explain its closer alignment with the BR classification, based exclusively on data from the Brazilian population.^8^ ^,^ ^10^ ^,^ ^19^

Substantial agreement for the classification of overweight (κ=0.811) and obesity (κ=0.795) between the IOTF and BR classification systems was reported by Freitas et al.^20^ in Brazilian children aged 6-10 years, supporting the findings. However, Silva et al.^21^ observed greater agreement in the prevalence of overweight between the BR and WHO in adolescents aged 14-19 years from Southern Brazil, whereas the IOTF classification showed the lowest agreement. It is hypothesized that discrepancies in agreement among classification systems within the Brazilian population may arise from the considerable ethnic, cultural, and economic diversity of a country with continental dimensions.

Regarding the near-perfect agreement between the IOTF and WHO, the WHO is based on a statistical model using data from the United States (US) for the development of its growth curves, which differs from the method used by the IOTF. Nevertheless, the statistical procedures applied allowed the curves to be smoothed and made more comparable across populations, even though they were derived exclusively from US children and adolescents included in the joint WHO-National Center for Health Statistics (NCHS) 1977 reference.^22^ ^,^ ^23^ The fact that the IOTF curves were developed using representative data from six countries, including Brazil and the United States, enables this classification to approximate both the WHO and the BR, reinforcing the excellent agreement observed in the study.

However, studies have not found excellent agreement between the IOTF and WHO classifications. An evaluation of the WHO, IOTF, and CDC classification systems in Mexican children and adolescents showed variation in the prevalence of weight ­categories across international references, with lower agreement between the IOTF and WHO.^22^ Conversely, data from a US cohort assessed using the same classification demonstrated that all classification systems showed good, although not excellent, agreement.^23^ These findings underscore the importance of assessing these systems across different adolescent populations in order to identify the most appropriate reference.

Although the WHO growth curves are widely used as a reference for assessing nutritional status in Brazil, differences in the populations used to construct these growth references may contribute to variations in overweight and obesity prevalence estimates compared with other classification systems.^19^ ^,^ ^21^ Despite the excellent agreement between the BR and WHO, the lower weighted kappa value suggests more pronounced differences in the BMI cutoff points used by these methods, which directly affect nutritional diagnosis. Developed exclusively with data from the Brazilian population, the BR classification may better reflect the nutritional status of Brazilian adolescents and offers greater precision in identifying overweight, isolated obesity, and CMR.^19^ ^,^ ^24^ Consequently, its practical application in clinical settings and school-based health programs could enhance the early identification of at-risk individuals, allowing for more timely and culturally appropriate nutritional interventions.

Nevertheless, the prevalence of obesity observed in the sample was even lower than that reported in other national studies. The Study of Cardiovascular Risk in Adolescents (ERICA) identified a national obesity prevalence of 8.4% and a prevalence of 7.4% for the Northeast region alone.^25^ These values are similar to the prevalence of 9.6% reported by Rodrigues et al.^26^ for a Northeastern capital but differs from the 6.3% prevalence found in the present study. This variation in obesity prevalence may be explained by differences in anthropometric assessment methods, sociodemographic characteristics of the study samples, and the location and period of data collection.

The study also identified an association between CMR and all overweight and obesity classifications evaluated. A substantial number of studies have highlighted the relationship between overweight and obesity in adolescents and the increase in physiological conditions that impair cardiometabolic profiles.^27^ ^,^ ^28^ To date, no study has compared the performance of a Brazilian classification with international classifications in detecting CMR, underscoring the relevance of the findings.

This comparative approach offers considerable applicability for both clinical practice and public health policy. Clinically, these findings can guide healthcare professionals in selecting the most appropriate classification for early and accurate identification of adolescents at increased cardiometabolic risk, enabling more precise and timely interventions. From a public health perspective, this research provides important evidence for refining national screening protocols and informing the development of context-specific strategies to effectively address and prevent cardiometabolic diseases among Brazilian adolescents.

Despite scientific evidence demonstrating the link between BMI and excess weight in adolescents,^29^ the wide variety of classifications used to assess BMI limits comparability across different populations.^24^ Therefore, there is a need for easily applicable indicators capable of screening the risk of cardiometabolic diseases while ensuring comparability across different pediatric populations. In this context, the results not only support the development of public health policies but also contribute to the alignment of global strategies aimed at controlling CMR in adolescents.

Among the adolescents assessed, the prevalence of CMR was higher when the BR classification was applied, which reinforces that its population-specific cut-off points are better suited to the nutritional profile of Brazilian adolescents. Although no studies have evaluated the performance of BR in detecting CMR compared with other classifications, its higher prevalence of overweight and obesity diagnoses has been previously reported, supporting its greater accuracy for this population.^19^

Furthermore, the prevalence of CMR was higher across all classifications when the diagnosis referred specifically to obesity, compared without excess weight and overweight. This finding reinforces the importance of detecting severe obesity, given that this condition is positively associated with poorer CMR profiles in adolescents.^7^ The association between obesity and CMR is observed across different stages of adolescence and is dependent on the degree of obesity, sex, and the specific cardiovascular factors assessed.^30^

Due to the lack of information on certain variables related to cardiometabolic metabolism, such as blood pressure, blood glucose, and insulin levels, the combination of dyslipidemia and WHtR (surrogate for IR) was used as indicators to assess CMR. Furthermore, because this is a cross-sectional study, the effectiveness of the classification systems used in predicting cardiometabolic outcomes in adulthood cannot be assessed. It is also important to note that the sample was representative of adolescents enrolled in a single Brazilian city, which limits the generalizability of the results to other Brazilian regions or educational contexts.

The greater sensitivity of the national classification in detecting CMR, revealed through this comparative approach, provides strong support for the development of guidelines and public policies aimed at controlling cardiovascular risk factors in youth. It is therefore suggested that additional nationwide studies be conducted to reinforce the applicability of these findings and enhance their representativeness for the Brazilian population.

The results show that the IOTF classification exhibited excellent agreement with the other references evaluated. In contrast, the BR classification of overweight and obesity demonstrated lower agreement with the WHO and a higher prevalence of CMR among adolescents assessed, reinforcing the distinction between classifications based on national *versus* international reference populations.

Given the growing obesity epidemic, particularly during childhood and adolescence, and its relationship with the development of cardiometabolic diseases in adulthood, the BR classification emerges as a valuable tool to optimize early detection of CMR in Brazilian health services. This, in turn, may facilitate timely interventions and support the prevention of cardiometabolic conditions in this population.

## Data Availability

The database that originated the article is available with the corresponding author.
